# Role of the horizontal gene exchange in evolution of pathogenic Mycobacteria

**DOI:** 10.1186/1471-2148-15-S1-S2

**Published:** 2015-02-02

**Authors:** Oleg Reva, Ilya Korotetskiy, Aleksandr Ilin

**Affiliations:** 1 Bioinformatics and Computational Biology Unit, Biochemistry Department, University of Pretoria, South Africa; 2 Scientific Center for Anti-infectious Drugs, 84 Auezov Str, Almaty 050008, Kazakhstan

**Keywords:** *Mycobacterium*, horizontal gene transfer, virulence

## Abstract

**Background:**

*Mycobacterium tuberculosis *is one of the most dangerous human pathogens, the causative agent of tuberculosis. While this pathogen is considered as extremely clonal and resistant to horizontal gene exchange, there are many facts supporting the hypothesis that on the early stages of evolution the development of pathogenicity of ancestral Mtb has started with a horizontal acquisition of virulence factors. Episodes of infections caused by non-tuberculosis Mycobacteria reported worldwide may suggest a potential for new pathogens to appear. If so, what is the role of horizontal gene transfer in this process?

**Results:**

Availing of accessibility of complete genomes sequences of multiple pathogenic, conditionally pathogenic and saprophytic Mycobacteria, a genome comparative study was performed to investigate the distribution of genomic islands among bacteria and identify ontological links between these mobile elements. It was shown that the ancient genomic islands from *M. tuberculosis *still may be rooted to the pool of mobile genetic vectors distributed among Mycobacteria. A frequent exchange of genes was observed between *M. marinum *and several saprophytic and conditionally pathogenic species. Among them *M. avium *was the most promiscuous species acquiring genetic materials from diverse origins.

**Conclusions:**

Recent activation of genetic vectors circulating among Mycobacteria potentially may lead to emergence of new pathogens from environmental and conditionally pathogenic Mycobacteria. The species which require monitoring are *M. marinum *and *M. avium *as they eagerly acquire genes from different sources and may become donors of virulence gene cassettes to other micro-organisms.

## Background

*Mycobacterium tuberculosis *is one of the most dangerous human bacterial pathogens, causing a potentially deadly disease that has been around for a long time. [1]. Despite of an optimistic report that tuberculosis and AIDS death rates are steadily declining around the world over recent several years [2], they remain the main killers. Moreover, HIV patients are much more vulnerable to tuberculosis and often become carriers for other non-tuberculous mycobacterial pathogens, such as *M. avium *[3-5], *M. kansasii *[4,5], *M. abscessus *[5], *M. timonense *[6] and *M. genavense *[7]. In the future these new pathogens may undergo the same evolutionary process of pathogenicity formation that was assumed for *M. tuberculosis *[8]. According to this hypothesis the evolution has started with an expansion phase involving active horizontal acquisition of virulence factors and gene duplication.

In the work by Reva & Bezuidt [9] a new channel of transfer of virulence genes from pathogenic Enterobacteria to *Brucella*, *Mycobacterium *and *Nocardia *was reported, which may pose a serious impact on emergence of new pathogens. Genomic islands found in *Mycobacterium *were most likely originated from alpha-Proteobacteria [10] and gamma-Proteobacteria [11]. An unexpectedly high frequency of mercury-resistant strains showing also an increased tolerance to gentamicin, streptomycin and D-cycloserine has been reported among the clinical non-tuberculous mycobacteria isolates of species *M. avium*, *M. intracellulare *and *M. scrofulaceum *[12]. The genome of a fish pathogen *M. marinum*, which sometimes causes opportunistic infections in humans, comprises a 23 kb mercury-resistance plasmid pMM2329. BLASTn and oligonucleotide composition comparison showed that this plasmid comprising a mercury resistance operon had originated from either *Nocardia *or *Pseudonocardia *[9]. It looked like that this plasmid has been acquired by *M. marinum *quite recently as it still shows a strong sequence and oligonucleotide pattern similarities to *Nocardia*. These newly acquired genes may be behind the increased drug resistance reported for *M. marinu *isolates [13]. Mercury resistance plasmid similar to that of *M. marinum *together with multiple GIs of *Pseudomonas *and *Actinobacteria *origin were identified in *M. abscessus*, a pseudotuberculous lung disease causing microbe [14]; and in a frog pathogen *M. ulcerans*, which sometimes cause skin ulcers in human [15].

In this work we analysed acquired genes and patterns of distribution of genomic islands in available genomes of Mycobacteria by comparing complete genome sequences and sequences of genomic islands previously identified in these organisms. The aim was to study the possibility of emergence of new mycobacterial pathogens in result of acquiring of virulence genomic islands.

## Results and discussion

**Figure 1:**
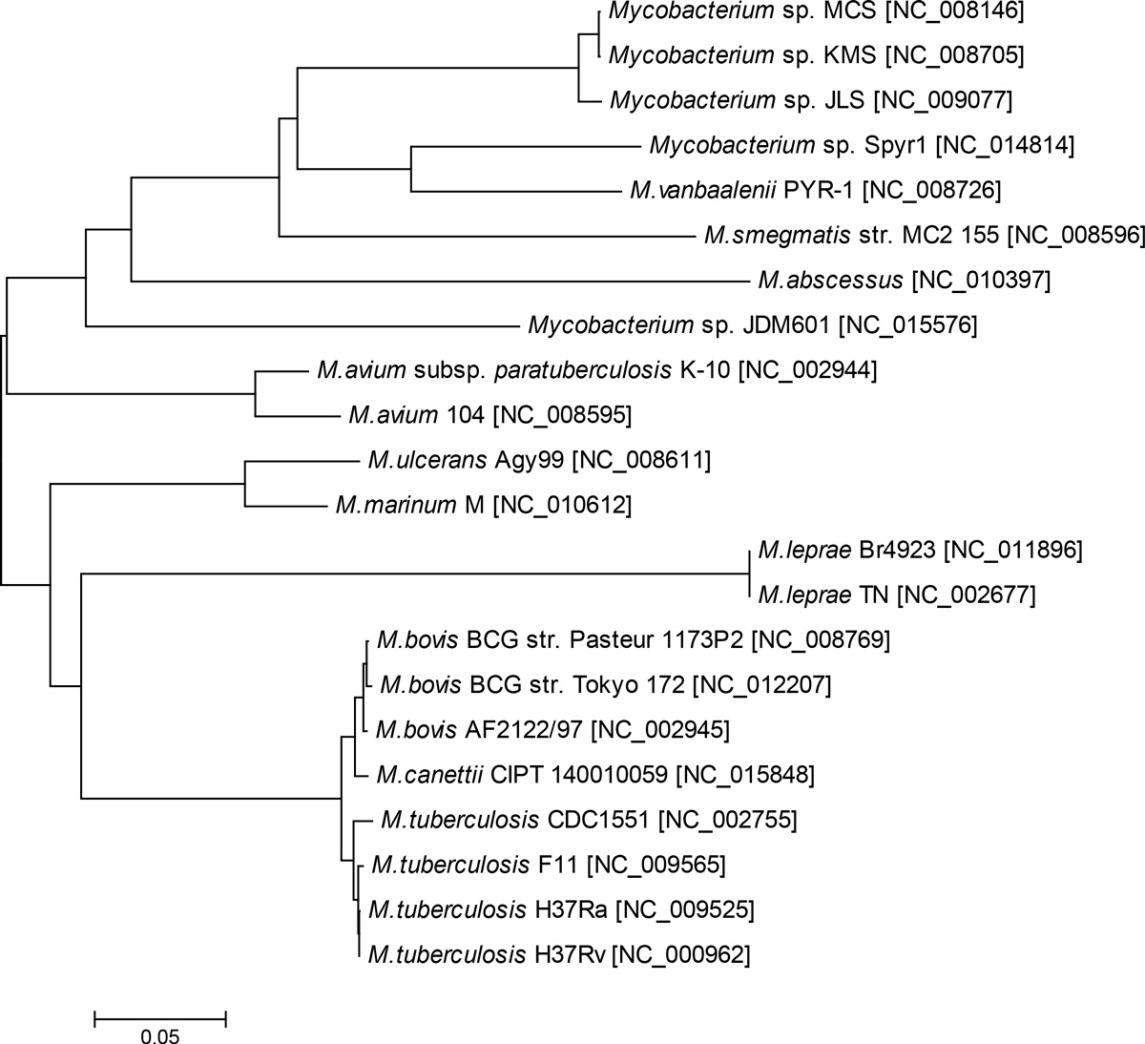
**Species phylogenetic tree**. The tree was constructed by Neighbour-Joining algorithm on concatenated alignments of 2,337 COGs.

Comparison of 22 mycobacterial genomes (Table 1) revealed 2,337 clusters of orthologous genes (COGs) shared by all these organisms. Concatenated alignment of these proteins was 657,505 amino acid residues long. A species tree inferred from the concatenated alignment is shown in Figure 1.

**Table 1:** Micobacterial genomes and genomic islands used in this study.

Genome and NCBI accession	Number of genomic islands	Total number of genes in genomic islands
*M. abscessus *[NC_010397]	10	300
*M. avium *104 [NC_008595]	20	538
*M. avium *ssp. *paratuberculosis *K-10 [NC_002944]	11	230
*M. bovis *AF2122/97 [NC_002945]	11	212
*M. bovis *BCG str. Pasteur 1173P2 [NC_008769]	10	187
*M. bovis *BCG str. Tokyo 172 [NC_012207]	11	209
*M. canettii *CIPT 140010059 [NC_015848]	11	194
*M. leprae *Br4923 [NC_011896]	22*	Not used
*M. leprae *TN [NC_002677]	23*	Not used
*M. marinum *M [NC_010612]	23	387
*M. smegmatis *MC2 155 [NC_008596]	12	329
*M. tuberculosis *CDC1551 [NC_002755]	10	203
*M. tuberculosis *F11 [NC_009565]	12	217
*M. tuberculosis *H37Ra [NC_009525]	12	225
*M. tuberculosis *H37Rv [NC_000962]	9	170
*M. ulcerans *Agy99 [NC_008611]	3	44
*M. vanbaalenii *PYR-1 [NC_008726]	16	360
*Mycobacterium *sp. JDM601 [NC_015576]	6	172
*Mycobacterium *sp. JLS [NC_009077]	14	430
*Mycobacterium *sp. KMS [NC_008705]	15	445
*Mycobacterium *sp. MCS [NC_008146]	12	356
*Mycobacterium *sp. Spyr1 [NC_014814]	14	419

* These genomic islands were excluded from this study.

**Figure 2:**
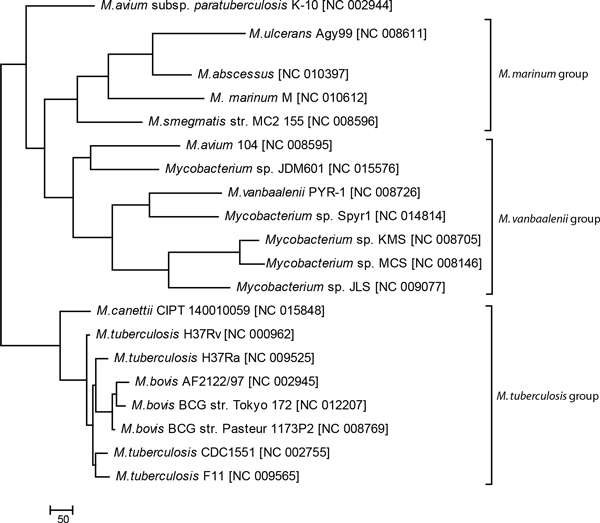
**Accessory gene tree**. Clustering of Mycobacterial genomes by sharing the accessory genes associated with genomic islands was performed by Wagner parsimony algorithm.

242 Genomic islands identified in 20 genomes (excluding two *M. leprae *genomes, see discussion in the 'Methods' section) comprised 5,627 genes, which formed 1,563 COGs. A binary data table representing the presence and absence of orthologous accessory genes associated with different genomic islands was created for inferring a parsimony tree shown in Figure 2.

In the tree in Figure 2 the clusters represent groups of organisms, which share the same pool of interchangeable mobile genetic elements. Species of the *M. tuberculosis *group were clustered separately from other mycobacteria in both trees in Figure 1 and 2. Finding of genomic islands in these micro-organisms contributed to the hypothesis by Veyrier *et al*. [8] that the pathogen evolution might be triggered out by the acquisition of horizontally transferred genes. However, all genomic islands in *M. tuberculosis *most likely are ancient acquisitions. The identification of relative time of insertion is grounded in the assumption that the process of amelioration alters the island nucleotide composition from the time of insertion to reconcile with that of the host in which it occurs [9]. In an interactive Web-based network of genomic islands prepared for our previous publication [16] the relative age of acquisition is depicted by grey gradient where the darker colour means recent acquisitions and lighter colours indicates ancient islands.

In total 48 genes were found which were associated exclusively with these genomic islands of the Mtb cluster and which were not present in genomic islands of other Mycobacteria. Among them there were *argFGHR *arginine biosynthesis operon; PE-PGRS family genes; *lpqD *lipoprotein; *idsB *and *grcC2 *genes involved in terpenoid biosynthesis; *mscL *osmotic pressure regulator; *moaB2 *stress response regulator and several hypotheticals.

Another group of micro-organisms clustered around *M. marinum *comprised phylogenetically related *M. ulcerans *and more distant *M. abscessus *and *M. smegmatis*. Hypothesis of sharing of common mobile genetic elements by these bacteria is supported by the fact that *M. marinum*, *M. ulcerans *and *M. abscessus *genomes contained almost identical plasmids with several virulence genes [13-15]. There were 5 hypothetical genes (17 genes when *M. smegmatis *is excluded), which were unique for the genomic islands of this group of micro-organisms. The third group clustered around *M. vanbaalenii *consisted of multiple environmental Mycobacteria. They shared 20 hypothetical genes unique for this group.

The strain *M. avium *subsp. *paratuberculosis *was located apart from other groups (Figure 2) and far away on the tree from its closest relative *M. avium *104 (compare to Figure 1). Presence of multiple genomic islands in *M. avium *subsp. *paratuberculosis *was confirmed by alternative genomic island prediction methods, as it was shown in Pre_GI database. Genetic content of the genomic islands and their ontological links to genomic islands from other micro-organisms were summarized in Additional file 1 supplementary Table S1.

From Additional file 1 Table S1 it was seen that the genomic islands of *M. avium *K-10 shared sequences with those from *M. avium *104, but according to Figure 2 the genetic content is rather different. Several genomic islands showed sequence similarity to rather distant genomic islands from *Mycobacterium canettii *and *Alicycliphilus denitrificans*. *M. avium *subsp. *paratuberculosis *is a causative agent of Johne's disease in cattle and other ruminants [17]. The non-paratuberculosis strain *M. avium *104 also was isolated from an adult AIDS patient, but this micro-organism was considered as an opportunist rather than an established pathogen [18]. It looks that the horizontal gene transfer was the driving force of evolution of the paratuberculosis lineage of *M. avium*, as regarding to other proteins both these subspecies are very much similar (Figure 1).

**Figure 3:**
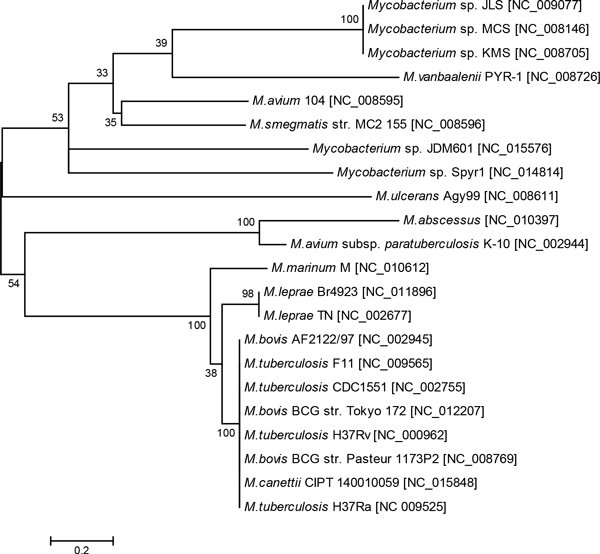
**FadD22 gene tree**. This dendrogram was inferred by Neighbour-Joining algorithm based on alignment of protein sequences of FadD22 found in different Mycobacterial genomes.

An interesting finding was that all these mycobacterial genomes possessed several common genes present in all species, which nevertheless were associated with horizontally transferred mobile genetic elements. These genes were *fadD22*-*fadE*, which are important for virulence and mycobactin synthesis [19,20], and also the O-succinylbenzoic acid-CoA ligase *menE *involved in menaquinone biosynthesis and considered as a potential target for antibiotics [21]. To ensure that these genes were associated with the horizontal gene transfer and were not falsely predicted due to some peculiarities in their sequences, a tree was constructed based on an alignment of the FadD22 proteins (Figure 3).

**Figure 4:**
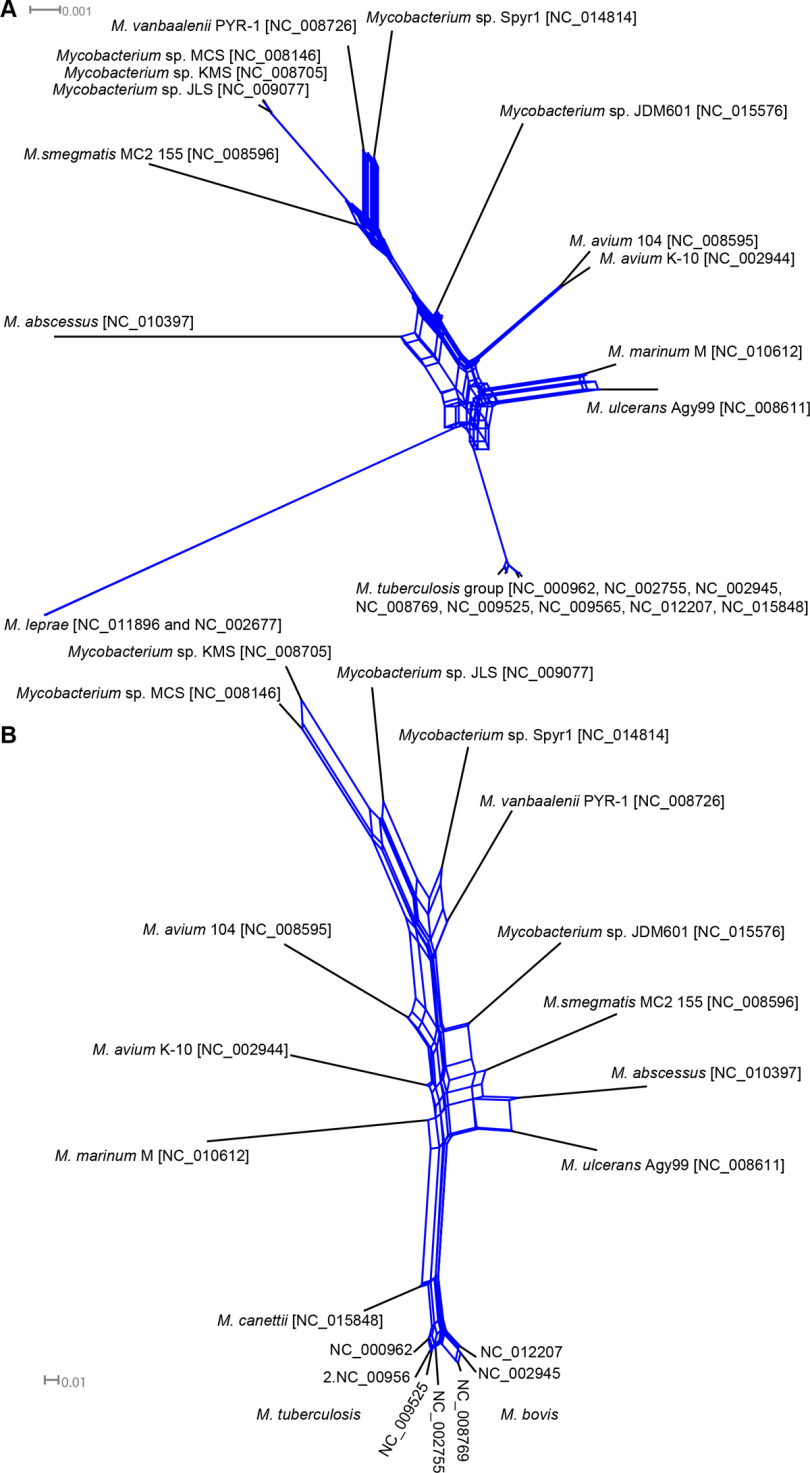
**Reticulate networks**. These networks demonstrate the events of exchange of A) core genes and B) accessory horizontally transferred genes between Mycobacteria. Reticulate events are depicted by blue lines.

A combination of high level variability of FadD22 across Mycobacteria with its conservation within taxonomic units indicates crucial importance of this protein for bacteria. FadD22 proteins showed much higher conservation in *M. tuberculosis *and *M. leprae *than that observed for other orthologous proteins (compare Figure 1 and 3) implying an indispensability of this protein for the pathogenesis. FadD22 of *M. marinum *also belonged to the Mtb group despite that this organism phylogenetically is quite distant from *M. tuberculosis*. Two strains of *M. avium *K-10 and 104 were separated in the FadD22 tree the same like in the genomic island gene tree (see Figure 2). In *M. avium *subsp. *paratuberculosis *K-10 this protein was similar to that from the pathogenic strain *M. abscessus*, while in *M. avium *104 the protein FadD22 was similar to orthologs in environmental strains. In general the topology of the FadD22 tree was not exactly congruent to either the species tree (Figure 1) or the accessory gene tree (Figure 2). It implies existing of a complex network of gene exchange between Mycobacteria. Two reticulate networks were designed by using the program SplitsTree: the first was based on incongruences of 2,337 individual core COG alignments (Figure 4A); and the second was based on the matrix of shared 1,563 COGs of horizontally transferred genes (Figure 4B).

The network of core genes (Figure 4A) showed that the species of *Mycobacterium *were quite isolated from each other and the individual gene trees in most cases were congruent to the species tree shown in Figure 1. Exceptions were *M. marinum *clustered with *M. ulcerans *in one case, and *M. vanbaalenii *clustered with *Mycobacterium *sp. Spyr1 in another case, which apparently have exchanged the core genes frequently. The turnover of genomic islands, which usually is associated with sharing the same pool of mobile genetic vectors such as conjugative plasmids and phages, was more intensive than the core gene exchange, especially between environmental *M. vanbaalenii *and *Mycobacterium *sp. Surprisingly, another common pool of mobile genes was shared by conditionally pathogenic *M. abscessus *and *M. ulcerans *with saprophytic soil *M. smegmatis *and *Mycobacterium *sp. JDM601. Although bacteria of the *M. tuberculosis *group were believed to be resistant to horizontal gene transfer and comprised only ancient genomic islands [9,22], it was still possible to root them to the common pool of mycobacterial horizontally transferred genes. Contrary, *M. avium *and *M. marinum *genomes were placed apart in the reticulate network. The reason for this might be that they acquired genes from different sources including those which were not common to other Mycobacteria (see Additional file 1 Table S1).

## Conclusions

Veyrier *et al*. [8] hypothesized that *M. tuberculosis *had undergone a biphasic evolutionary process involving genome expansion (gene acquisition and duplication) and reductive evolution (deletions). Nowadays the evolution of this pathogen including the development of drug resistance fully relies on selective mutations, genome recombination and gene duplication [22], but the evolution towards pathogenicity initially might be triggered by an acquisition of several virulence factors [9]. Over the recent decades the humankind has witnessed a drastic emergence of outbreaks of new pathogens. A question of an acute medicinal importance is where, when and which pathogens may cause new outbreaks in the near future? Drawing the strongest attention to control on *M. tuberculosis*, we have not to forget that other non-tuberculosis Mycobacteria have a potential to put humankind under risk of new invasions. This is why it is very important to study in detail the development of pathogenicity of *M. tuberculosis *so that an emergence of new mycobacterial pathogens will not catch us unaware.

Genomic islands found in Mycobacteria share DNA composition and sequence similarities with a big group of genomic islands originated from Actinobacteria, alpha-, beta- and gamma-Proteobacteria, *Deinococcus*/*Thermus *and some other bacteria [23] (see also the online interactive network of genomic islands at [16]). In the same paper it was shown that the genomic islands of *M. tuberculosis *most likely have originated from alpha-Proteobacterial intracellular parasites and symbionts of *Agrobacterium*, *Rhizobium *and *Brucella *genera. An activation of genetic vectors of this group was reported and it was hypothesized that it might be resulted from up-growing ocean water pollution with heavy metal ions and other industrial pollutants [9,24]. According to Bezuidt *et al*. [24], the recent outbreak of the enterohemorrhagic *E. coli *O104:H4 in 2011 was associated with an activation of this pool of mobile virulence genes. The same virulence vectors may affect in future the environmental and conditionally pathogenic Mycobacteria. Potentially the most risky species in this regard are *M. marinum *(fish pathogen) and *M. avium *ssp. *paratuberculosis *(cattle pathogen) as i) they were promiscuous in acquiring mobile genetic elements from different sources including taxonomically distant organisms; and ii) *M. marinum *might actively exchange genes with other environmental and conditionally pathogenic species of *Mycobacterium*. The latter capability is potentially dangerous as the exchange of readily available virulence genes between compatible potentially pathogenic bacteria may lead to spontaneous stochastic outbreaks of new diseases.

## Methods

### Genomes and annotation data used in this research

Complete genome sequences of 22 strains of Mycobacteria were obtained from NCBI database [25] in genbank format. Genomic island data including the annotation of all associated genes were obtained from Pre_GI database [26]. Numbers of genomic islands and horizontally acquired genes per genome are summarized in Table 1.

Genomic islands stored in Pre_GI were identified by SeqWord Gene Island Sniffer (SWGIS) program [27,28]. The analysis of gene content of genomic islands showed that the predicted genomic loci in *M. leprae *contained many unexpected conserved core genes like *dnaA *replication helicase and gyrase sub-units. These genomic islands most likely were false predicted resulting from a degeneration of the genome specific pattern of biased frequencies of oligonucleotides probably due to a higher rate of mutations [27]. Extremely high level of compositional variability of *M. leprae *genomes was confirmed by genome visualization using SWGIS [29,30]. According to Pre_GI data, the multiple genomic islands identified by SWGIS in the *M. leprae *genomes were not confirmed by other programs (IslandViewer and PAGIDB). To avoid further false predictions, the genomic islands of *M. leprae *were excluded from consideration in this research.

### Phylogenomic inferences

Clusters of orthologous genes (COG) were identified by BLASTp alignment of all genes from different genomes against each other. Pairs of genes in two genomes where considered as orthologs if they reciprocally returned the best BLASTp hits. On the next step the MUSCLE alignment [31] was used to filter out those BLASTp predictions where the alignment coverage was less than 70% of the length of aligned proteins. Resulting alignment files were used for designing gene trees, but prior to phylogenetic analysis every alignment file was edited by the program Gblocks to remove ambiguous blocks [32]. For phylogenetic inferences based on alignments of orthologous sequences the super-matrix and super-tree approaches were used. In the former case all alignments were concatenated sequentially into an artificial super-alignment that was then analysed by the Neighbour-Joining algorithm implemented in MEGA6 [33]. In the latter case the phylogenetic trees were inferred individually for every COG alignment by the Neighbour-Joining algorithm implemented in neighbor.exe executable file of the PHYLIP package and then all the gene trees were reconciled into a reticulate phylogenetic network by the program SplitsTree [34].

A phylogenetic tree based on the presence and absence of accessory genes associated with the genomic islands was inferred by using the Wagner parsimony algorithm in pars.exe executable file of the PHYLIP package.

## List of abbreviations

COGs - clusters of orthologous genes;

SWGIS - SeqWord Genomic Island Sniffer;

Mtb - *Mycobacterium tuberculosis*.

## Competing interests

The authors declare that they have no competing interests.

## Authors' contributions

OR - bioinformatics support, programming, manuscript preparation;

IK and AI contributed equally to data validating and manuscript preparation.

## Supplementary Material

Additional file 1 Genomic islands of *M. avium* subsp. *paratuberculosis* K-10 Click here for file

## Declarations

Publication of this article has been funded by the program "Study on the reversion of antibiotic resistance in pathogenic microorganisms" funded in Kazakhstan (#0113PК00831). Programming and bioinformatics research was funded by the National Research Foundation of South Africa (#86941).

This article has been published as part of *BMC Evolutionary Biology *Volume 15 Supplement 1, 2015: Selected articles from the IX International Conference on the Bioinformatics of Genome Regulation and Structure\Systems Biology (BGRS\SB-2014): Evolutionary Biology. The full contents of the supplement are available online at http://www.biomedcentral.com/bmcevolbiol/supplements/15/S1.
