# Supplementary material for: Role of the horizontal gene exchange in evolution of pathogenic Mycobacteria
Source: BMC Evol Biol. 2015 Feb 2;15(Suppl 1):S2. doi: 10.1186/1471-2148-15-S1-S2 (PMC4331801; doi:10.1186/1471-2148-15-S1-S2)
Supplement: Additional file 1 — Genomic islands of M. avium subsp. paratuberculosis K-10 [file 1471-2148-15-S1-S2-S1.docx]

Supplementary Table 1. Genomic islands of *M. avium* subsp. *paratuberculosis* K-10.

| Location in genome | Gene content summary | Best BLASTn hit to genomic islands in other species | Possible donor organism* |
| --- | --- | --- | --- |
| 99000..117073 | Hypotheticals | *Mycobacterium avium* 104 | *Pseudonocardia* |
| 292974.. 314208 | Hypotheticals; osmoprotection proteins | *M. avium* 104 | *Pseudonocardia* |
| 865425.. 899160 | Hypotheticals, Fad-proteins | *M. avium* 104 | *Pseudonocardia*, *Geodermatophilus*, *Frankia*, *Beutenbergia* |
| 1285436.. 1321324 | Hypotheticals; esterase; GDP-D-mannose dehydratase; nucleotide-sugar epimerase; drug resistance | *Mycobacterium canettii* | *Pseudonocardia* |
| 1781905.. 1803222 | Hypotheticals; alpha1,3-glucosyltransferase; isocitrate lyase | *Alicycliphilus denitrificans* | *Pseudonocardia* |
| 1881405.. 1904640 | Hypotheticals; hydrolases; membrane proteins | *M. avium* 104 | *Pseudonocardia* |
| 2373873.. 2399346 | Hypotheticals; IS-element; heat-inducible transcription repressor; phosphohydrolase | *M. avium* 104 | *Pseudonocardia* |
| 4147431.. 4173538 | Hypotheticals | *M. avium* 104 | *Pseudonocardia* |
| 4163490..4199728 | Hypotheticals; IS-element; membrane proteins | *M. avium* 104 | *Pseudonocardia* |
| 4254011.. 4277488 | Hypotheticals; deoxycytidine triphosphate deaminase | *Mycobacterium marinum* | *Blastococcus* |
| 4446083.. 4467623 | Hypotheticals; pyrroline and glutamyl reductase; phosphoserine phosphatase; dehydratase | *Mycobacterium liflandii* | *Pseudonocardia* |

*Donor organisms are given as predicted in Pre_GI database.
